# Functional status and health-related quality of life following Young and Burgess classified pelvic ring injuries

**DOI:** 10.1371/journal.pone.0346671

**Published:** 2026-04-09

**Authors:** Camryn C. Therrien, Myrthe Uil, Kaj ten Duis, Hester Banierink, Anne M. L. Meesters, Nymke M. Trouwborst, Anette J. Hamminga, Jean-Paul P. M. de Vries, Frank F. A. IJpma, Inge H. F. Reininga

**Affiliations:** 1 Department of Trauma Surgery, University Medical Center Groningen, University of Groningen, Groningen, The Netherlands; 2 Department of Surgery, University Medical Center Groningen, University of Groningen, Groningen, The Netherlands; Fudan University, CHINA

## Abstract

**Purpose:**

To determine recovery rates of patient-reported outcome measures (PROMs) in terms of functional status and health-related quality of life (HR-Qol) compared to the general population, as well as mortality rates and reintervention rates in patients with Young and Burgess (YB) classified pelvic ring injuries.

**Methods:**

This cohort included patients with YB classified pelvic ring injuries in a level 1 trauma centre between 2007 and 2024. Dutch Short Musculoskeletal Function Assessment subscales were used to evaluate functional status, and the Dutch version of the EuroQol-5D 5L was used to assess HR-Qol. Outcomes were compared to normative data of the Dutch population. Patient-reported outcomes, mortality, and reintervention rates were reported for each YB classification. Full recovery was defined as reaching the lower level of the 95% confidence interval of the normative data in all outcome measures.

**Results:**

PROMs were available for 346 (75%) of the eligible patients. The median follow-up time was 2.2 (IQR 3.6) years. The following rates of full recovery were observed: lateral compression (LC)1 = 41%, LC2 = 46%, LC3 = 11%, anterior posterior compression (APC)1 = 36%, APC2 = 33%, APC3 = 40%, vertical sheer (VS)=31% and combined mechanical injury (CM) =27%. The highest 30-day mortality rates were found in LC3 (19%), APC3 (16%), and VS (12%) injuries. LC3, APC3, VS, and CM injuries had the highest reintervention rates due to pain or discomfort.

**Conclusion:**

Patients with LC1, LC2, APC1, APC2, and APC3 had comparable outcomes to the general population, while LC3, VS, and CM injuries had worse outcomes. LC3, APC3, and VS injuries had the highest mortality rates within 30 days of the initial trauma. LC3, APC3, VS, and CM had slightly higher levels of treatment revisions or removal of osteosynthesis material.

## Introduction

Pelvic ring injuries have an annual incidence of 14–37 out of 100,000 inhabitants [1,2] and can result in significant morbidity or mortality [3–9]. These injuries occur in an extremely heterogeneous group of patients, ranging from young patients who endure high-energy traumas such as motor vehicle or cycling accidents to elderly patients who sustained injuries from low-energy falls [1,2,10]. The Young and Burgess (YB) classification was developed to better understand the pelvic injury patterns and level of injury instability. This was initially done in the early work of Pennal et al. and later modified by Tile et al. [11,12]. Young and Burgess then further developed this classification system [13,14], which stratifies injuries into groups based on the direction of force applied to the pelvis during the trauma and, ultimately, the osseous and ligamentous injury patterns observed on radiographic imaging [14]. The classifications included are lateral compression (LC), which is further divided into LC1, LC2, and LC3; anterior-posterior compression (APC), divided into APC1, APC2, and APC3; vertical shear (VS); and combined mechanical injury (CM). The YB classification system is widely used in clinical practice, as it enables quick and accurate assessment of the injury and can be used to guide treatment decisions [2,15–18].

Although patient-reported outcome measures (PROMs) following pelvic ring injuries have been examined in several studies [3–5,7,19,20], no study to date has reported PROMs or recovery rates for each specific YB classified pelvic ring injury. Describing outcomes for specific YB classifications would enable surgeons to better inform patients about the expected recovery from their injury, particularly given the widespread use of the YB classification system in clinical practice.

Accordingly, this study aims to gain insight into the functional status and health-related quality of life in patients who endured the various YB classified pelvic ring injuries. The primary research question is: 1) To what extent do patients with pelvic ring injuries, classified by the YB classification system, recover in terms of patient-reported functional status and quality of life, compared to the general population?; and the secondary research questions are: 2) What is the mortality rate in each YB injury classification group? And 3) What is the reintervention rate in each YB injury classification group?

## Methods

### Study design

In this cohort, data from both a retrospective study (4), which included patients from 03-03-2007–31-12-2016 (with data being extracted on 01-01-2017), and a prospective study (5), which included patients from 01-01-2017–31-01-2025, were combined. The analyses were started on 01-02-2025.

These studies took place in a level 1 trauma centre and referral centre for all pelvic injuries in the northern part of the Netherlands. All patients above 18 years of age, without cognitive disorders, and who could read and understand Dutch were informed about the study and asked to participate by completing questionnaires. In the retrospective cohort study, patients who had reached at least one year of follow-up were asked to complete the questionnaires once. Patients included in the prospective cohort study were asked to complete questionnaires at several timepoints: at admission (recalled pre-injury) and six weeks, three months, six months, one year, two years, and five years following the injury. The latest available follow-up moment available for each patient was used, with a minimum follow-up time of one year. Consequently, outcomes of patients who died within one year of the injury could not be included in the PROMs analysis.

The local Medical Ethical Review Board reviewed the methods and waived further need for approval (METc 2016.385 and METc 2017.543). For patients included in the retrospective follow-up study, written informed consent was obtained. For patients included in the prospective follow-up study, verbal and written informed consent was provided by all patients, witnessed by the treating surgeon, and documented in the electronic patient files. All data collected was pseudo-anonymized and was not directly traceable to the patients.

### Data collection

Patient information was collected by reviewing each patient’s electronic medical and surgical records. The patient and injury characteristics collected included gender, age, whether it was an isolated pelvic ring injury (no additional injuries sustained), associated lower extremity injuries, high or low energy trauma, survival in the first 30 days and the first year following the injury, and whether the patient was treated non-operatively or operatively. Additionally, surgical reconstruction methods were recorded. The Dutch Trauma Registry provided data regarding the Injury Severity Score (ISS) [21], which was used to categorise trauma as minor (ISS < 16) or severe (ISS ≥ 16).

Initial radiographs were verified by two pelvic trauma surgeons, and the injuries were classified according to the YB classification system [14]. Patients with pelvic injuries that were not classifiable according to the YB classification system were not included in this study, for example, isolated pubic ramus or iliac fractures that maintain an intact posterior arch or isolated transverse fractures of the sacrum (S3, S4, S5) or coccyx [2,22].

### Patient outcomes

#### Patient-reported outcome measures.

The Dutch Short Musculoskeletal Function Assessment (SMFA-NL) was used to evaluate patient-reported functional status. This questionnaire comprises 46 items, each answered on a five-point Likert scale. From these items, specific subscales can be derived. The subscales used in this study were: lower extremity dysfunction (LED), difficulties with daily activities (ADL), and mental and emotional problems (MEP), all of which have demonstrated structural validity [23–25]. The individual item scores were summed and transformed into a total score ranging from 0 to 100, with higher scores indicating better functional status.

To assess patient-reported health-related quality of life, the Dutch version of the EuroQol-5D 5L (EQ-5D) questionnaire was administered. This instrument is recognised as a valid and reliable instrument and is used to evaluate health-related quality of life across five key dimensions: mobility, self-care, routine activities, pain/discomfort, and anxiety/depression [26,27]. Each dimension was answered on a five-point Likert scale, generating a utility score between 0 and 1, where higher scores indicate a better outcome.

Normative data of the SMFA-NL [28] and the EQ-5D [26] were used to determine whether the patient recovered to the expected level of their age group in the general Dutch population. SMFA-NL normative data were further specified by gender. If the patient’s score reached the lower level of the 95% confidence interval (CI) of the normative data, the patient was considered recovered. A full recovery was defined by a patient having recovered in the three SMFA-NL subscales and the EQ-5D.

#### Mortality.

Mortality was recorded for all patients with a YB classified pelvic ring injury, irrespective of consent for participation in retrospective or prospective follow-up. Consequently, patients who died prior to enrolment were able to be included in the analysis. This outcome was grouped into mortality within 30 days, between 30 days and 1 year, and after 1 year following the injury. Whether the trauma was the cause of mortality was recorded.

#### Treatment revision.

The need for treatment revision, in terms of reintervention for operatively treated patients, delayed intervention for the initially conservatively treated patients, or removal of osteosynthesis material due to infection or discomfort, was recorded as an outcome in all classification groups.

### Statistical analysis

Statistical analyses were conducted using IBM SPSS software with a significance level of p < 0.05. Normally distributed data were presented as means and standard deviations (SD), while non-normally distributed data was presented as medians and interquartile ranges (IQR). Frequency was reported as n (%). Results were presented collectively for all patients, stratified by YB injury type.

A non-response analysis was performed to determine differences in patient and injury characteristics between responders and non-responders. For categorical variables, a Pearson Chi-Square test was conducted, and for continuous variables, the Mann-Whitney U test was used.

Patient outcomes and recovery rates were analysed using descriptive statistics and presented in a table. The Mann-Whitney U test was conducted to assess if there were differences between the PROMs scores of the study population and the normative data from the Dutch population.

Lastly, a sub-analysis of PROMs of patients treated operatively vs conservatively was conducted using the Mann-Whitney U test.

## Results

### Study population

A total of 1014 patients presented with a pelvic ring injury between 2007 and 2024 in our hospital. Of these, 38.6% (n = 391) could not be classified using the YB classification system. The remaining 623 patients were classifiable, with the majority (51%) having LC1 injuries. Patient and injury characteristics for the entire study group, as well as for each classification subgroup, are presented in Table 1.

**Table 1:** Patient characteristics.

	All YB injuries n = 623 (100%)	LC1 n = 317 (51%)	LC2 n = 57 (9%)	LC3 n = 69 (11%)	APC1 n = 22 (4%)	APC2 n = 56 (9%)	APC3 n = 32 (5%)	VS n = 50 (8%)	CM n = 20 (3%)
Age, median (IQR)	53 (38)	55 (37)	56 (44)	44 (52)	41 (41)	56 (27)	44 (37)	48 (31)	54 (25)
Age > 65 years, n (%)	186 (30)	102 (32)	23 (40)	24 (35)	4 (18)	13 (23)	4 (13)	11 (22)	5 (25)
Female, n (%)	274 (44)	168 (53)	19 (33)	35 (51)	8 (36)	5 (9)	9 (28)	21 (42)	9 (45)
HET, n (%)	464 (75)	215 (68)	45 (79)	57 (83)	19 (86)	47 (84)	28 (88)	41 (82)	12 (60)
Fragility fractures*, n (%)	97 (16)	61 (19)	10 (18)	11 (16)	0	5 (9)	1 (3)	5 (10)	4 (20)
ISS ≥ 16, n (%)	238 (38)	95 (30)	21 (37)	32 (46)	6 (27)	24 (43)	17 (53)	31 (62)	12 (60)
ICU admittance, n (%)	213 (34)	82 (26)	17 (30)	32 (46)	7 (32)	20 (36)	18 (56)	26(52)	11 (55)
Days in ICU, median (IQR)	5 (11)	5 (11)	6 (17)	5 (10)	2 (3)	6 (12)	5 (14)	3 (10)	7 (24)
Isolated pelvic ring injury, n (%)	176 (28)	99 (31)	15 (26)	17 (25)	9 (41)	17 (30)	5 (16)	9 (18)	5 (25)
Lower extremity injuries, n (%)	143 (23)	51 (16)	17 (30)	23 (33)	6 (27)	15 (29)	9 (28)	14 (28)	8 (40)
Operative treatment, n (%)	217 (35)	45 (15)	15 (26)	35 (51)	8 (36)	42 (75)	24 (75)	35 (70)	13 (65)
Pelvic external fixator, n (%)	77 (12)	10 (3)	4 (7)	16 (23)	2 (9)	15 (27)	10 (31)	16 (32)	4 (20)
Anterior only fixation, n (%)	56 (9)	12 (4)	2 (4)	8 (12)	8 (36)	17 (30)	6 (19)	1 (8)	1 (10)
Posterior only fixation, n (%)	48 (8)	14 (4)	2 (4)	10 (15)	0	5 (9)	4 (13)	8 (16)	5 (25)
Anterior & posterior fixation, n (%)	97 (16)	17 (5)	9 (16)	14 (20)	0	18 (32)	12 (25)	22 (44)	5 (25)

*Fragility fractures are defined as injuries caused by a low-energy trauma in patients above the age of 65.

Young and Burgess (YB), lateral compression (LC), anterior-posterior compression (APC), vertical shear (VS), combined mechanical injury (CM), high-energy trauma (HET), injury severity score (ISS), intensive care unit (ICU).

### Availability of patient-reported outcome measures

**Fig 1:**
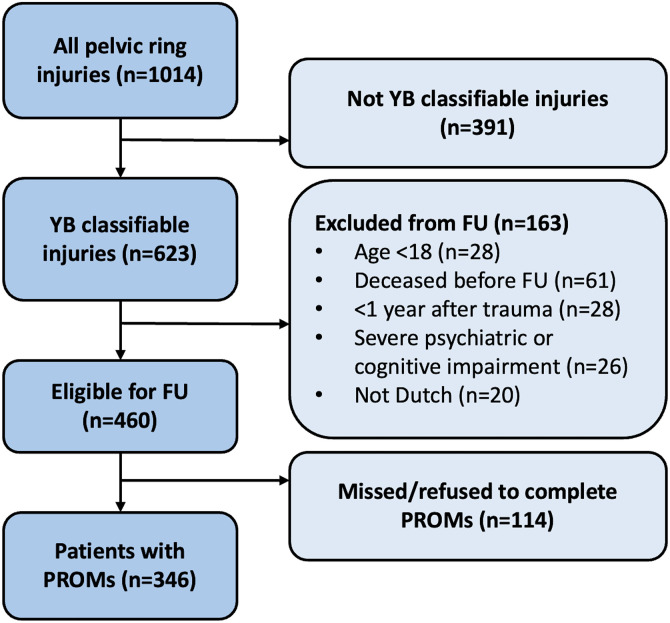
Flowchart depicting the availability of patient-reported outcome measures (PROMs).

A flowchart illustrating patient inclusion and availability of PROMs is presented in Fig 1. Of the 623 patients with YB classified injuries, 460 (74%) were deemed eligible to participate in filling out PROMs. Among those eligible, 346 patients (75%) completed PROMs, with a median follow-up duration of 2.2 years (IQR 3.6).

A non-response analysis identified a higher prevalence of isolated pelvic ring injuries (p = 0.04) in non-responders compared to responders (S1 Appendix). No other differences were identified.

### Patient-reported outcome measures and recovery rates of Young and Burgess injuries

**Fig 2:**
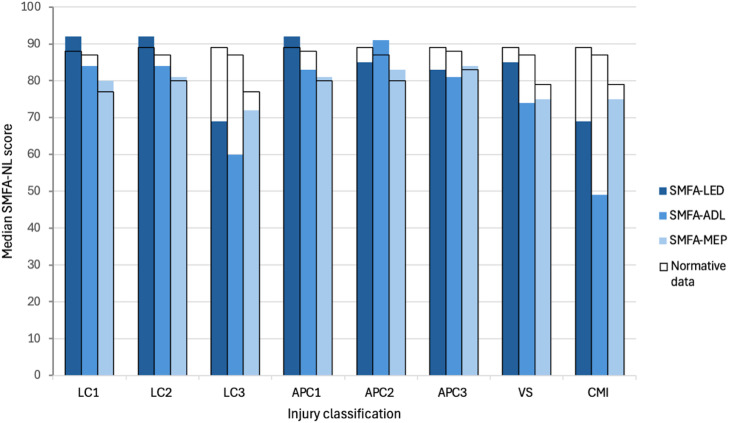
Median Dutch Short Musculoskeletal Function Assessment (SMFA-NL) subscale scores (lower extremity dysfunction (LED), difficulties with daily activities (ADL), mental and emotional problems (MEP)) for all injury classifications: lateral compression (LC), anterior-posterior compression (APC), vertical shear (VS), combined mechanical injury (CM). The patient’s scores are indicated in blue, and the scores of the normative data of the uninjured peers are outlined in black.

**Fig 3:**
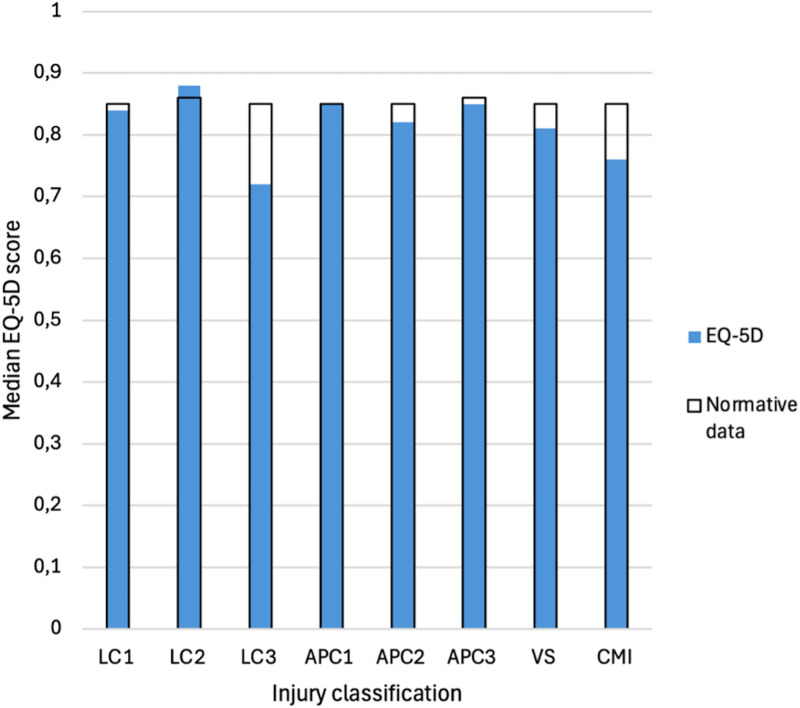
Median scores of the Dutch version of the EuroQol-5D 5L (EQ-5D) for all injury classifications: lateral compression (LC), anterior-posterior compression (APC), vertical shear (VS), and combined mechanical injury (CM). The patient’s scores are indicated in blue, and the scores of the normative data of the uninjured peers are outlined in black.

**Fig 4:**
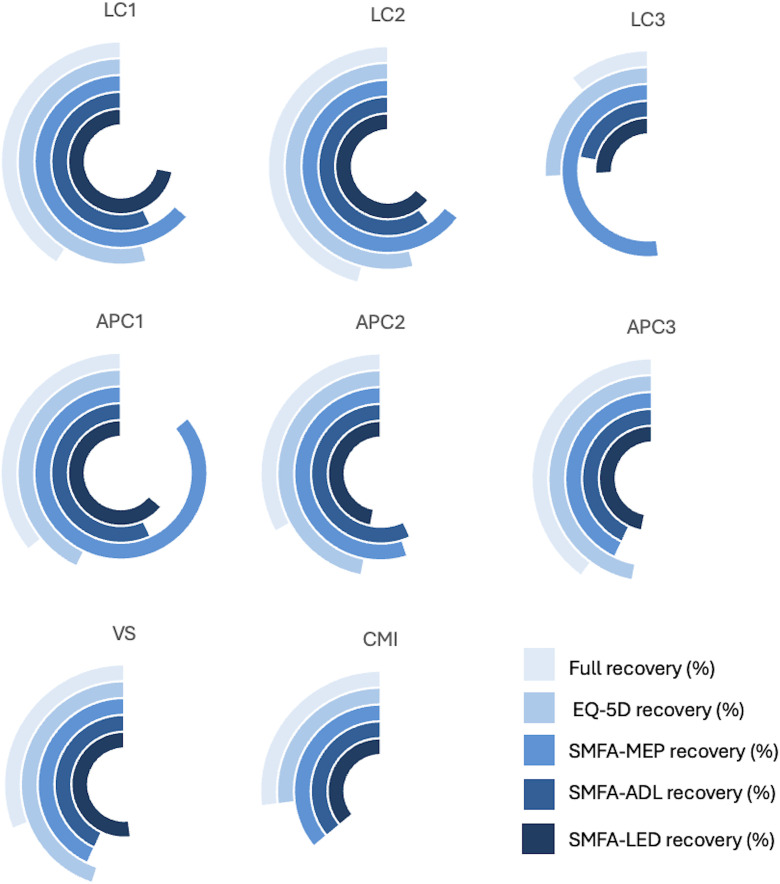
Recovery rates for each Young and Burgess classification: lateral compression (LC), anterior-posterior compression (APC), vertical shear (VS), combined mechanical injury (CM) for a full-recovery, recovery of Short Musculoskeletal Function Assessment (SMFA) subscale scores (lower extremity dysfunction (LED), difficulties with daily activities (ADL), mental and emotional problems (MEP)) and the Dutch version of the EuroQol-5D 5L (EQ-5D). A full circle represents 100% recovery.

Table 2 presents the median SMFA-NL subscales and EQ-5D scores for all patients, as well as outcomes for the individual YB classifications and normative data from the Dutch population. Significant differences between patient outcomes and normative data per group are highlighted in bold in Table 2. The median scores of the patients and the age and gender matched normative data are graphically presented in Fig 2 and Fig 3. Furthermore, recovery rates of the individual PROMs and a full recovery are presented in Fig 4 for each injury classification. Patients with LC1, LC2, APC1, APC2, and APC3 injuries demonstrated functional outcomes and health-related quality of life comparable to those of the general Dutch population. The full recovery rates of these patients were 41%, 46%, 36%, 33% and 40%, respectively. The classifications with statistically significantly worse outcomes compared to their peers in the general population were LC3, VS, and CM injuries. Similarly, these injuries also showed the lowest levels of recovery, with 11%, 31%, and 27% fully recovered, respectively (Fig 4). A sub-analysis of full recovery rates for subgroups (high-energy traumas, age > 65, operatively treated patients, and isolated pelvic injuries) is provided in S2 Appendix. Furthermore, the sub-analysis of operatively vs conservatively treated patients showed no statistically significant difference in PROMs (S3 Appendix).

**Table 2:** SMFA-NL subscale scores and EQ-5D outcomes compared to normative data from the Dutch population.

	All YB injuries with PROMs (n = 346)	LC1 (n = 194)	LC2 (n = 26)	LC3 (n = 27)	APC1 (n = 14)	APC2 (n = 30)	APC3 (n = 15)	VS(n = 29)	CM (n = 11)
Follow-up in years, median (IQR)	2.2 (4)	2.5 (4)	2.1 (3)	2.0 (4)	4.3 (4)	2.1 (3)	2.2 (4)	2.0 (3)	1.1 (4)
**SMFA LED**									
Median (IQR)	89 (25)	92 (19)	92 (19)	69 (30)	92 (24)	85 (21)	83 (27)	85 (36)	69 (35)
Dutch population, median (IQR)	89 (6)	88 (4)	89 (2)	89 (10)	89 (3)	89 (2)	89 (6)	89 (6)	89 (6)
P-value*	0.88	**0.008**	0.63	**0.008**	0.70	0.52	0.39	0.23	**0.028**
Patients recovered, n (%)	206 (61)	134 (72)	16 (64)	7 (26)	9 (64)	14 (47)	7 (47)	15 (52)	4 (36)
**SMFA ADL**									
Median (IQR)	81 (34)	84 (31)	84 (35)	60 (38)	83 (27)	91 (31)	81 (32)	74 (44)	49 (43)
Dutch population, median (IQR)	87 (9)	87 (9)	87 (2)	87 (12)	88 (8)	87 (1)	88 (6)	87 (9)	87 (9)
P-value*	**0.001**	0.64	0.26	**<0.001**	0.23	0.42	0.25	**0.022**	**0.019**
Patients recovered, n (%)	177 (52)	109 (57)	15 (60)	6 (22)	8 (57)	17 (57)	6 (43)	12 (43)	4 (36)
**SMFA MEP**									
Median (IQR)	78 (25)	80 (28)	81 (27)	72 (31)	81 (13)	83 (24)	84 (31)	75 (3	75 (34)
Dutch population, median (IQR)	79 (3)	77 (8)	80 (7)	77 (8)	80 (3)	80 (3)	83 (4)	79 (8)	79 (8)
P-value*	0.98	0.31	0.55	0.09	0.45	0.56	0.65	0.15	0.15
Patients recovered, n (%)	208 (61)	122 (64)	17 (65)	14 (52)	12 (86)	17 (55)	6 (43)	12 (43)	4 (36)
**EQ-5D**									
Median (IQR)	0.83 (0.26)	0.84 (0.23)	0.88 (0.20)	0.72 (0.20)	0.85 (0.16)	0.82 (0.14)	0.85 (0.14)	0.81 (0.22)	0.76 (0.29)
Dutch population, median (IQR)	0.85 (0.01)	0.85 (0.01)	0.86 (0.05)	0.85 (0.05)	0.85 (0.07)	0.85 (0.02)	0.86 (0.07)	0.85 (0.01)	0.85 (0.01)
P-value*	**<0.001**	0.14	1.00	**<0.001**	0.35	0.27	0.10	**0.013**	**0.008**
Patients recovered, n (%)	169 (49)	105 (54)	14 (54)	7 (26)	6 (43)	14 (47)	7 (47)	13 (45)	3 (27)
Patients fully recovered**									
**n (%)**	128 (37)	80 (41)	12 (46)	3 (11)	5 (36)	10 (33)	6 (40)	9 (31)	3 (27)

*The P-value indicates a significant difference in PROMs compared to the normative data. Significance was set as a p-value of <0.05.

**Patients were considered fully recovered when the scores on the three SMFA-NL subscales, as well as the EQ-5D, fell above the lowest limit of the 95% CI of the normative data.

Young and Burgess (YB), lateral compression (LC), anterior-posterior compression (APC), vertical shear (VS), combined mechanical injury (CM), Short Musculoskeletal Function Assessment (SMFA), lower extremity dysfunction (LED), difficulties with daily activities (ADL), mental and emotional problems (MEP), the Dutch version of the EuroQol-5D 5L (EQ-5D).

### Mortality

**Fig 5:**
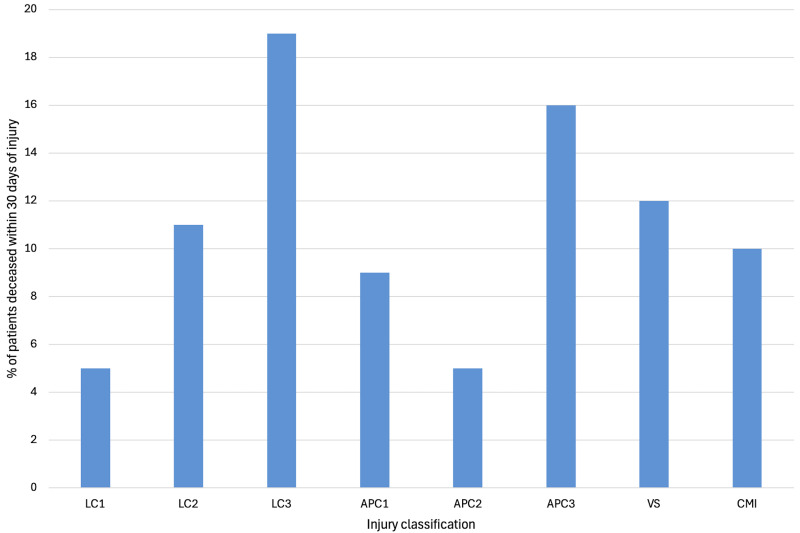
Mortality within 30 days of the injury for each injury classification: lateral compression (LC), anterior-posterior compression (APC), vertical shear (VS), combined mechanical injury (CM).

Table 3 and Fig 5 present the 30-day mortality rate for each YB classification. Of the 53 patients who were deceased within 30 days, 45 (85%) were due to the trauma, and 8 (15%) were due to other causes. The classification with the highest mortality rate within 30 days of the trauma was the LC3 injuries (19%), followed by the APC3 injuries (16%) and the VS injuries (12%).

**Table 3:** Mortality rate per injury classification.

	All YB injuries n = 623	LC1 n = 317	LC2 n = 57	LC3 n = 69	APC1 n = 22	APC2 n = 56	APC3 n = 32	VS n = 50	CM n = 20
Deceased within 30 days, n (%)	53 (9)	16 (5)	6 (11)	13 (19)	2 (9)	3 (5)	5 (16)	6 (12)	2 (10)
Deceased between 30 days and 1 year, n (%)	11 (2)	5 (2)	4 (7)	0	0	0	2 (6)	0	0
Deceased after 1 year, n (%)	34 (5)	17 (5)	6 (11)	3 (4)	1 (5)	4 (7)	0	3 (6)	0

Young and Burgess (YB), lateral compression (LC), anterior-posterior compression (APC), vertical shear (VS), combined mechanical injury (CM**).**

### Treatment revision

Three out of 272 (1%) patients with LC1 injuries who were originally treated conservatively required delayed operative intervention, all approximately one year following the injury (Table 4). Some LC3 (n = 1, 1%), VS (n = 1, 2%) and CM (n = 2, 10%) injuries that were treated operatively required surgical revision. In all injury classifications, at least one patient required the removal of osteosynthesis material either due to infection or discomfort. Reintervention due to implant failure, including the recession of screws or breakage of plates, occurred most frequently in APC1 (n = 2) and APC2 (n = 2) injuries.

**Table 4:** Treatment revision per injury classification.

	All YB injuries n = 623	LC1 n = 317	LC2 n = 57	LC3 n = 69	APC1 n = 22	APC2 n = 56	APC3 n = 32	VS n = 50	CM n = 20
Delayed intervention, n (%)	3 (0)	3 (1)	0	0	0	0	0	0	0
Operative treatment revision, n (%)	3 (0)	0	0	1 (1)	0	0	0	1 (2)	2 (10)
Removal of OSM due to infection, n (%)	12 (2)	3 (1)	1 (2)	0	0	1 (2)	3 (9)	3 (6)	1 (5)
Removal of OSM due to discomfort, n (%)	21 (3)	4 (1)	2 (2)	2 (3)	1 (5)	1 (2)	3 (9)	5 (10)	3 (13)
Reintervention due to implant failure, n (%)	8 (1)	2 (1)	0	1 (1)	2 (9)	2 (4)	0	1 (2)	0

Young and Burgess (YB), lateral compression (LC), anterior-posterior compression (APC), vertical shear (VS), combined mechanical injury (CM), osteosynthesis material (OSM).

## Discussion

This study provides patient-reported functional status and health-related quality of life for each YB classified pelvic ring injury. Patients with LC1, LC2, APC1, APC2, and APC3 injuries demonstrated functional outcomes and health-related quality of life comparable to those of the general Dutch population at least one year post-injury, with 41%, 46%, 36%, 33% and 40%, respectively, achieving full recovery. In contrast, patients with LC3, VS, and CM injuries experienced significantly worse outcomes, including greater lower extremity dysfunction, more difficulty with activities of daily living, and a lower overall quality of life, with 11%, 31%, and 27%, respectively, reaching a full recovery. Furthermore, it was identified that LC3, APC3, and VS injuries had the highest mortality rates within 30 days of the initial trauma, and that LC3, APC3, VS, and CM had slightly higher levels of treatment revisions or removal of osteosynthesis material due to pain or discomfort.

### Patient-reported outcomes

While several studies have examined the patient-reported outcomes following pelvic ring injuries [3–5,7,19,20,29–32], to our knowledge, this is one of the first to report specific outcomes for each YB injury classification. Bott et al. incorporated the YB classification in their analysis of predictors for poor PROMs (Short Form-36 and EQ-5D-3L) following pelvic ring injuries, finding no differences in outcomes between YB injuries [29]. However, their analysis was restricted to the broader categories of LC, APC, and VS injuries, without differentiating between specific subtypes. This may have obscured meaningful differences, as our study identified substantial variation in outcomes, particularly between LC1 and LC3 injuries. These findings underscore the importance of analysing injury subtypes individually rather than grouping them into broader categories, which risks concealing clinically relevant differences in patient outcomes. Additionally, Bott et al. only presented patient-reported outcomes scores for the entire cohort, not specific to each classification group. These limitations may be attributed to their small sample size, comprised of 36 LC, 18 APC, and 20 VS injuries, compared to 247, 58, and 29 with PROMs in our study.

Moreover, Gabbe et al. reported the number of patients who were living independently and who had returned to work within the LC, APC, and VS/CM categories [33]. However, similar to Bott et al., injuries were grouped into broad categories without distinguishing between specific subtypes. As a result, their analysis did not reveal significant differences in outcomes between YB classifications. This further highlights the importance of analysing outcomes by specific injury subtypes to avoid obscuring clinically relevant differences.

Similarly, Suzuki et al. also found no significant differences in outcomes (Majeed score, the Iowa Pelvic Score, and the Medical Outcomes Study Short-Form 36) between YB classification groups [30]. However, only the APC and VS classifications were included in their analysis, and they did not provide specific outcomes for each classification. Their cohort also had a limited sample size with 38 LC, 12 APC, 6 VS, and 1 CM.

Our findings have important implications for both future research and clinical practice, as a valuable reference point for future research aiming to evaluate or compare pelvic ring injury outcomes is provided. Furthermore, since the YB classification system is widely used in clinical practice and communication among trauma and orthopaedic surgeons, having classification-specific outcome data enables clinicians to offer patients more accurate, personalised information regarding expected recovery trajectories. This allows patients to be provided with realistic expectations on recovery and guides rehabilitation planning. For instance, being able to communicate that patients with LC1 injuries generally experience outcomes comparable to the general population in all domains, while those with LC3 may face more significant long-term limitations in terms of lower extremity dysfunction, daily living and health-related quality of life. Moreover, we observed that relatively few patients achieved full recovery across all domains. This highlights not only the immense long-term impact of pelvic ring injuries on physical functioning and quality of life, but also the need to improve postoperative care and rehabilitation strategies to better support patients in reaching complete recovery.

### Mortality

Several studies have previously explored the relationship between YB classification and outcomes such as haemorrhage, transfusion requirements, associated injuries, and mortality [34–42]. In our cohort, the highest 30-day mortality rates were observed among patients with LC3 (19%), APC3 (16%), and VS (12%) injuries. These findings align with existing literature [35,37,39], which supports the notion that more severe pelvic ring injuries, as classified by the YB system, are associated with increased mortality. Mortality is often due to associated injuries sustained during the trauma, rather than to the pelvic ring injury itself.

Accordingly, our analysis found that patients with LC1 and APC1 injuries more often sustained isolated pelvic trauma, whereas LC3 and APC3 injuries were frequently accompanied by additional injuries. This trend corresponds with earlier studies showing that injury severity and concomitant injuries increase with YB classification grade [35,39,40,42]. Specifically, Manson et al. reported that more unstable injuries (LC2, LC3, APC3, VS, CM) were associated with higher transfusion requirements and an increased incidence of concomitant injuries compared to LC1 and APC1 injuries [35]. Similarly, Dalal et al. reported that LC3 injuries were associated with higher rates of pelvic vascular injury, retroperitoneal hematoma, circulatory shock, and fluid requirements compared to LC1 injuries [39]. Additionally, APC3 injuries were linked to greater involvement of visceral organs, pelvic vascular injury, and compromised haemodynamic status compared to APC1 injuries [39]. Starr et al. also noted more severe chest trauma and higher mortality rates in LC3 patients compared to those with LC1 injuries [42].

While our study did not evaluate specific concomitant injuries or haemodynamic status, we included other measurements of injury severity, such as ICU admission and Injury Severity Score (ISS) of ≥16. The frequency of ICU admission was more common, and an ISS ≥ 16 was the most frequent in LC3, APC3, VS, and CM injuries, which is also reflected in other studies [35,42].

### Surgical reintervention

To our knowledge, no previous studies have reported rates of surgical revision or implant removal for each YB injury type. In our cohort, LC3, APC3, VS, and CM injuries had the highest frequency of surgical reinterventions, including revision procedures and removal of osteosynthesis materials due to pain or discomfort. This is of importance, as it seems to coincide with the PROMs in these classification groups. Accordingly, revision surgery may contribute to longer recovery times, additional complications, and ultimately poorer functional outcomes. Although, this should be interpreted with caution due to the low number of reinterventions in all classification groups.

### Strengths and limitations

Several limitations of this study should be acknowledged. While the sample size is relatively large, the cohort was predominantly composed of patients with LC1 injuries, which may result in underrepresentation of other injury types. However, this distribution is consistent with trends reported in previous studies [14,29,30,42]. We also encountered two limitations related to the matching of normative data. Firstly, SMFA-NL normative data is not available for individuals over the age of 75. Consequently, all patients above 75 were matched to the 66–75-year age group, which may have led to an underestimation of recovery rates in those patients. Secondly, normative data for the EQ-5D outcome matched to both age and gender were not available. As a result, recovery in female patients may also be underestimated. Lastly, our non-response analysis revealed a slightly lower proportion of isolated pelvic ring injuries among PROM respondents. Though concomitant injuries frequently accompany pelvic ring injuries [30,35,42], their impact on patient-reported outcomes warrants consideration. Larger multicenter prospective studies are warranted to validate these findings and to improve the representation of patients with less commonly occurring injuries.

### Conclusion

LC1, LC2, APC1, APC2, and APC3 injuries present with similar levels of functional status and health-related quality of life at least one year following the injury, compared to the uninjured population. In contrast, patients with LC3, VS, and CM injuries experience poorer recovery, including greater lower extremity dysfunction, problems with daily living, and reduced quality of life. Furthermore, LC3, APC3, and VS injuries have the highest 30-day mortality rates, and LC3, APC3, VS, and CM show the highest rates of surgical revision or removal of osteosynthesis material due to pain or discomfort. As the YB classification system is widely used in clinical practice, this study provides information that can be used to inform patients on recovery trajectories and set realistic expectations for patients with specific injuries.

## Supporting information

S1 Appendix Non-response analysis. (DOCX)

S2 Appendix Subgroup analysis of each Young and Burgess classification that have fully recovered. (DOCX)

S3 Appendix Differences in PROMs for operatively vs conservatively treated patients in each Young and Burgess classification. (DOCX)

S1 Data Data set YB PLOS One. (XLSX)
