# Supplementary material for: Functional status and health-related quality of life following Young and Burgess classified pelvic ring injuries
Source: PLoS One. 2026 Apr 9;21(4):e0346671. doi: 10.1371/journal.pone.0346671 (PMC13065045; doi:10.1371/journal.pone.0346671)
Supplement: S1 Appendix — (DOCX) [file pone.0346671.s001.docx]

***Appendix 1*** *Non-response analysis*

|  | **Respondents (n= 346)** | **Non-respondents (n = 114)** | **p-value*** |
| --- | --- | --- | --- |
| **Age at injury, median (IQR)** | 55 (28) | 53 (41) | 0.41 |
| **Female, n (%)** | 154 (45) | 53 (46) | 0.71 |
| **ISS>16, n (%)** | 120 (35) | 33 (30) | 0,26 |
| **ICU admittance, n (%)** | 97 (28) | 32 (29) | 0.84 |
| **Lower extremity injuries, n (%)** | 66 (19) | 26 (23) | 0.39 |
| **Isolated pelvic ring injury, n (%)** | 103 (30) | 46 (40) | **0.04** |
| **Operative treatment, n (%)** | 132 (38) | 32 (28) | 0.05 |
| **YB classification, n (%)** |  |  | 0.98 |
| LC1 | 194 (56) | 60 (53) |  |
| LC2 | 26 (8) | 10 (9) |  |
| LC3 | 27 (8) | 12 (11) |  |
| APC1 | 14 (4) | 4 (4) |  |
| APC2 | 30 (9) | 11 (10) |  |
| APC3 | 15 (4) | 4 (4) |  |
| VS | 29 (8) | 9 (8) |  |
| CM | 11 (3) | 4 (4) |  |

**The P-value indicates a significant difference in responders and non-responders. Significance was set as a p-value of <0.05.*

Injury severity score (ISS), intensive care unit (ICU), Lateral Compression (LC), Anterior-Posterior Compression (APC), Vertical Shear (VS), Combined Mechanical Injury (CM), Young and Burgess (YB)
