# Supplementary material for: Functional status and health-related quality of life following Young and Burgess classified pelvic ring injuries
Source: PLoS One. 2026 Apr 9;21(4):e0346671. doi: 10.1371/journal.pone.0346671 (PMC13065045; doi:10.1371/journal.pone.0346671)
Supplement: S2 Appendix — (DOCX) [file pone.0346671.s002.docx]

**Appendix 2** Subgroup analysis of each Young and Burgess classification that have fully recovered

| **Subgroups** | **Fully recovered Y&B injuries with PROMs**  **(n=128)** | **Fully recovered LC1**  **(n=80)** | **Fully recovered LC2**  **(n=12)** | **Fully recovered LC3**  **(n=3)** | **Fully recovered APC1**  **(n=5_** | **Fully recovered APC2**  **(n=10)** | **Fully recovered APC3**  **(n=6)** | **Fully recovered VS**  **(n=9)** | **Fully recovered CM**  **(n=3)** |
| --- | --- | --- | --- | --- | --- | --- | --- | --- | --- |
| **High energy traumas,**  **n (%)** | 94 (38) | 55 (42) | 10 (46) | 2 (10) | 4 (36) | 9 (39) | 6 (43) | 2 (33) | 2 (33) |
| **Age >65,**  **n (%)** | 33 (32) | 18 (31) | 4 (57) | 1 (9) | 1 (50) | 5 (41) | 0 | 3 (38) | 1 (33) |
| **Operative patients,**  **n (%)** | 43 (32) | 15 (46) | 5 (55) | 1 (5) | 3 (60) | 6 (26) | 3 (33) | 8 (35) | 1 (13) |
| **Isolated pelvic injuries, n (%)** | 36 (34) | 24 (39) | 2 (67) | 1 (11) | 2 (25) | 4 (31) | 1 (100) | 2 (25) | 0 |

Injury severity score (ISS), intensive care unit (ICU), Lateral Compression (LC), Anterior-Posterior Compression (APC), Vertical Shear (VS), Combined Mechanical Injury (CM)
