# Supplementary material for: Functional status and health-related quality of life following Young and Burgess classified pelvic ring injuries
Source: PLoS One. 2026 Apr 9;21(4):e0346671. doi: 10.1371/journal.pone.0346671 (PMC13065045; doi:10.1371/journal.pone.0346671)
Supplement: S3 Appendix — (DOCX) [file pone.0346671.s003.docx]

**Appendix 3** Differences in PROMs for operatively vs conservatively treated patients in each Young and Burgess classification

|  | **All YB injuries with PROMs**  **(n=346)** | **LC1 (n=194)** | **LC2 (n=26)** | **LC3 (n=27)** | **APC1 (n=14)** | **APC2 (n=30)** | **APC3 (n=15)** | **VS (n=29)** | **CM (n=11)** |
| --- | --- | --- | --- | --- | --- | --- | --- | --- | --- |
| Operatively treated patients, n (%) | 132 (38) | 33 (17) | 9 (35) | 19 (70) | 5 (36) | 23 (77) | 12 (80) | 23 (79) | 9 (72) |
| SMFA LED | 0.060 | 0.888 | 0.569 | 0.221 | 0.140 | 0.571 | 0.612 | 0.317 | 0.260 |
| SMFA ADL | 0.072 | 0.821 | 0.571 | 0.158 | 0.350 | 0.325 | 0.464 | 0.300 | 0.184 |
| SMFA MEP | 0.419 | 0.532 | 0.481 | 0.079 | 0.347 | 0.606 | 0.464 | 0.626 | 0.412 |
| EQ-5D | 0.205 | 0.431 | 0.711 | 0.260 | 0.240 | 0.737 | 0.633 | 0.142 | 0.376 |
